# How forage grain ratoon rice improves the grain yield during the ratoon season

**DOI:** 10.3389/fpls.2024.1402677

**Published:** 2024-09-11

**Authors:** Panpan Gai, Yuanwei Chen, Xin Sun, Hongjing Chen, Desheng Yang, Miaofei Ren, Lei Liu, Weiqin Wang, Hua-bin Zheng, Qiyuan Tang

**Affiliations:** ^1^ College of Agronomy, Hunan Agricultural University, Changsha, Hunan, China; ^2^ Crop Research Institute, Hunan Academy of Agricultural Sciences, Changsha, China

**Keywords:** ratoon rice, regeneration rate, stubble height, nutrients, grain yield

## Abstract

**Introduction:**

In recent years, with the rapid expansion of ratoon rice production in Hunan, a unique ratoon rice-based production system, forage-grain ratoon rice (FG-RR), has been newly developed. Ratooning rice is a season of rice harvested by utilizing the dormant buds on the rice stubble left after harvesting the first season of rice to sprout and grow. Therefore, the characteristics of stalks themselves are crucial for the production of ratoon rice. The cutting period and higher stubble height directly affect the characteristics of ratoon rice stubbles. Based on this, we conducted the following research.

**Methods:**

In 2021 and 2022, field experiments were conducted in central China to study the effects of different cutting periods and stubble height on the regeneration rate and nutrient content of ratoon crops. The treatments included two cutting periods (10 days after heading in the first season and 30 days after heading in the first season, respectively referred to as T10 and T30) and two stubble heights (10 cm and 30 cm, respectively referred to as H10 and H30).

**Results:**

Compared with the T30H30 treatment, T10H10 and T10H30 increased grain yield by 48.1%, 41.7%, 73.1%, and 65.2% in the two-year ratoon seasons, while T30H10 reduced grain yield by 30.9% and 19.5% in the two years, respectively. Early cutting increased the panicles, spikelets per panicle, and filled grain rate to varying increase, while higher stubble height increased panicles but decreased spikelet panicle. On the one hand, early cutting and higher stubble height increased the dry and fresh weight, nonstructural carbohydrates (NSCs), organic carbon (C), and nitrogen content of rice stalks, thereby improving the regeneration rate of ratoon rice. On the other hand, early cutting and higher stubble height retention increased the accumulation of nitrogen in rice stubble throughout the entire growth period and facilitated the transport of nitrogen to the mature panicles.

**Discussion:**

Therefore, appropriate early cutting and higher stubble height retention are the keys to improving the grain yield and stability of ratoon rice.

## 1 Introduction

Feeding herbivores has become increasingly common in southern China. However, there are few extensive grassland areas, and the development of recycled forage has become an important agricultural technology. It has been reported that developing recycled rice for forage can increase forage production without creating additional demand on fields in subtropical and temperate rice areas. Still, it cannot balance cereal production with livestock feed production (Chen et al., 2022). Therefore, balancing rice and feed production has become the focus of our research (Dong et al., 2020). A large amount of ratoon forage was obtained by leaving stakes 30 cm from the first season harvest, spraying 60 g hm^−2^ erythromycin, and harvesting ratoon forage on the 37th day after the first season rice harvest while maintaining a harvest height of 5 cm. However, the quality of rice grain was poor in the first season and excellent in the regrowth season (Huang et al., 2020). The idea of obtaining high-quality rice grain in the ratoon season, supplemented by rice straw in the first season, was then switched to mowing at the pre-furrow period, which increased the ratoon grain yield to 7.5 t ha^−1^ (unpublished data from this group). Studies indicated that FG-RR systems have higher energy output and higher energy utilization efficiency, while also reducing the potential for global warming and eutrophication (Qi et al., 2024b). As such, FG-RR systems may comprise a novel approach to reconcile the simultaneous demands of grain and forage production on the same land parcel, although further work *in situ* is required to elucidate prospects associated with this claim.

The regeneration ability of ratoon rice determines the grain yield of the ratoon season. The regeneration rate can be used as an indicator to evaluate the regeneration ability of regenerated buds, and the level of regeneration ability ultimately determines the grain yield of regenerated buds. Regeneration ability is a complex trait determined by variety traits, climatic conditions, and agronomic measures (Bond and Bollich, 2006). Researchers have conducted extensive studies on different agronomic measures. Studies indicated that the dry weight of a single stem sheath during the first season of rice maturity has the greatest direct impact on regeneration ability (Ren et al., 2006). Higher stubble height increased the regeneration rate and grain yield of regenerated crops due to the increase in the weight of stubble (Takeo et al., 2017). In short, the higher stubble dry weight left after the first harvest provides more carbohydrates, which are beneficial for the growth of regenerated buds. The process of ratooning can be explained by apical dominance (Wang et al., 2020), which is a phenomenon in plants involving the inhibition of axillary bud outgrowth by the shoot apex. Regenerated crops, after removing their apical dominance, stored reserves when their photosynthetic products are insufficient to meet their own growth needs (White, 1973).Carbohydrate reserves are considered the substrate for plant growth and respiration. During the growth and development process of the first season, nonstructural carbohydrates accumulate in the leaves and stems of rice plants and are transported to the grains after flowering (Turner and Fund, 1993). Under normal mature conditions, when harvesting the first crop, the stubble does not accumulate NSC again (Yoshinaga et al., 2013). In the early growth period of ratoon rice, the germination and growth of regenerated tillers are mainly attributed to the nutrients stored in the first season stubble (He et al., 2019), and the nutrients stored in the stubble are positively correlated with regeneration ability (Santos et al., 2003). When the first crop season is harvested, the starch in the stubble is rapidly hydrolyzed and transported to new tillers (Slewinski, 2012). Xu and Xiong (2000) indicated that the increase in the non-structural carbohydrates of the rice stubble increased both generation speed and the total number of auxiliary buds during ratoon season. The stubble is the primary NSC storage organ in grasses. As the stubble height decreases, the NSC content shows a decreasing trend (Lee et al., 2008). The higher the dry weight of the first season rice after harvest, the more carbohydrates are available for the growth of regenerated buds.

The difference in harvest time in the first season has a more direct impact on the grain yield of regenerated crops, even surpassing the use of nitrogen fertilizer, with higher RC grain yield obtained when harvested before maturity in MC in Japan (Tanaka et al., 2021). After the first season of heading, a large number of regenerated buds die, mainly due to an increase in the amount of light energy allocated to the grains during the grain-filling period of the main crop, and a decrease in the amount of light energy allocated to the regenerated buds. On the 14th, 7th, and 0th day before harvest, the survival rates of regenerated buds treated with ear removal were 1.9% to 15.9%, 23.9% to 29.6%, and 34.8% to 51.1% higher than the control, respectively (Xu et al., 2021). Yang et al. (2022) also found that poor grain filling in the first season leads to a higher accumulation of nutrients such as carbohydrates and mineral elements in the stem, which also promotes the germination of regenerated buds. In short, the main reasons for the survival rate and length of regenerated buds depend on the biomass storage and light energy allocation in the stem sheath. Therefore, the agronomic measures that are beneficial for increasing the NSC content in stubble after the first harvest are worth exploring to promote the germination of regenerated shoots and increase the grain yield during the ratoon season. The height of stubble and harvesting time in the first season are two main agronomic measures to improve the dry weight and NSC content of stubble after harvesting in the first season.

Based on previous research on “forage-grain ratoon rice (FG-RR),” the first season of rice should be harvested 30 days after the full heading period, with a stubble height of 10 cm (Chen et al., 2022). At this time, the carbon footprint is small and the economic benefits are high. However, to achieve high grain yield in the regeneration season, it is crucial to understand how the mowing period and stubble height affect grain yield through the characteristics of the stubble. We speculate that: 1) early mowing and high stubble retention may increase the grain yield potential of RC; 2) the methods to increase RC grain yield may differ between early mowing and high stubble retention.

## 2 Materials and methods

### 2.1 Experimental site and design

**Figure 1:**
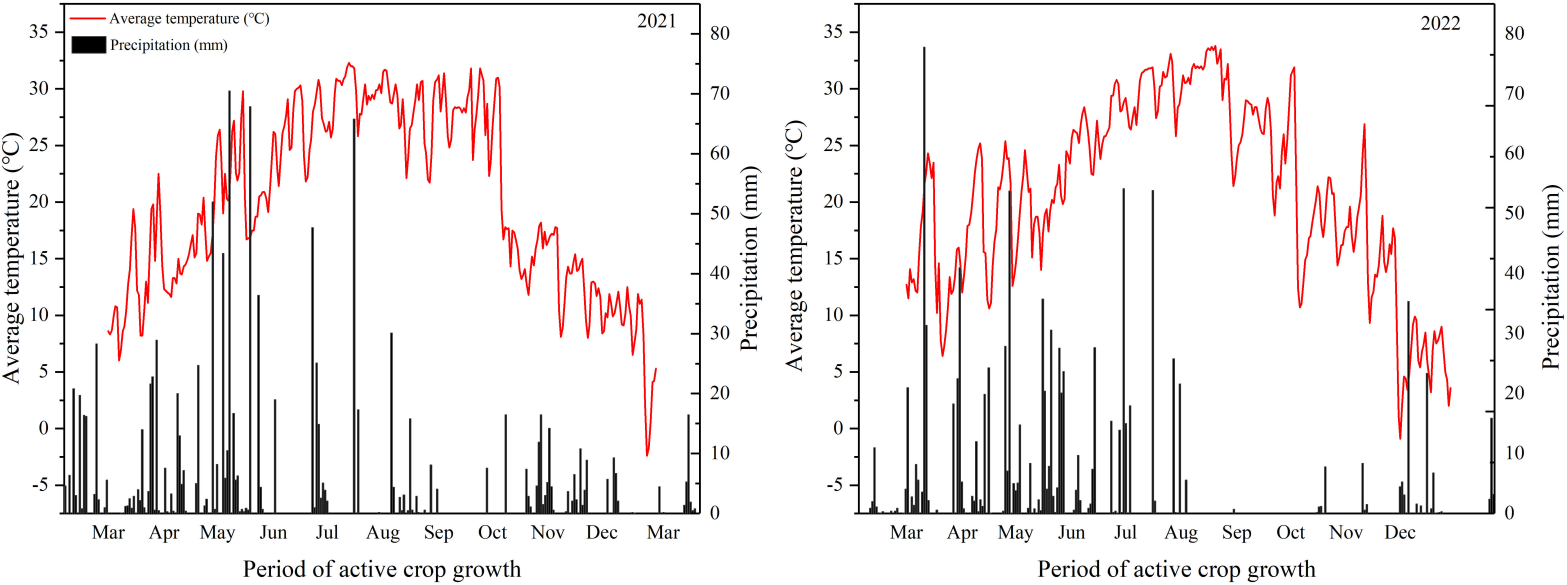
Average temperature and precipitation during the 2021–2022 rice growing season.

Field experiments were conducted during the rice growth seasons of 2021 and 2022 in Jinjing Town, Changsha County, Hunan Province, central and southern China (28° 53′ N, 113° 38′ E). The soil in the experimental field was acidic red soil evolved from granite, and the basic soil fertility over the past two years is shown in **Table 1**. During these two years, climate parameters, including daily minimum temperature, and maximum temperature were collected from a weather station (Em50, METER Group, Inc. USA) approximately 100 m away from the experimental field during the growth period from the transplanting of the main crop (MC) to the maturity of the ratoon crop (RC) (**Figure 1**).

**Table 1:** Soil nutrient status in the 0 cm–20 cm soil layer of the experimental field.

Year	pH	Organic matter (g kg^−1^)	Total nitrogen (g·kg^−1^)	Available phosphorus (mg·kg^−1^)	Available potassium (mg·kg^−1^)
2021	5.78	18.7	1.5	18.3	122.3
2022	5.53	19.9	1.8	18.9	117.7

The tested variety was Xiangliangyou 900 (XLY900). It is a super hybrid rice variety developed by Hunan Nianfeng Seed Industry Technology Co., Ltd. and Hunan Hybrid Rice Research Center using Guangxiang 24S × R900 breeding. According to previous experiments conducted by the research group, XLY900, as a type of “forage-grain ratoon rice,” showed better silage grain yield and quality in the first season and better grain yield and rice quality in the ratoon season (Chen et al., 2022). The experiment adopted a randomized block design with two mowing period treatments: 10 days after heading in the first season (T10) and 30 days after heading in the first season (T30); and two stubble height treatments: low stubble height 10 cm (H10) and high stubble height 30 cm (H30), repeated three times (**Table 2**). The plot size was 20 m^2^ (4 m × 5 m). To prevent the leakage of fertilizer and water between plots, a 30 cm field ridge was used to separate the areas, and the field ridges were covered with plastic film. For MC, a total of 195 kg N·hm^-2^ was applied three times, with basal fertilizer: tillering fertilizer: ear fertilizer = 5:2:3. For RC, a total of 150 kg N·hm^−2^ was applied twice, N_bud_:N_tiller_ = 5:5. All nitrogen fertilizers were applied in the form of urea. The application amount of P_2_O_5_ was 97.5 kg·hm^−2^ (all basal application in the first season), and the application amount of K_2_O was 240 kg·hm^−2^ (195 kg·hm^−2^ in the first season, basal fertilizer: ear fertilizer = 5:5; 45 kg·hm^−2^ in the ratoon season, all as seedling fertilizer).

**Table 2:** Description of different mowing periods and stubble height treatments.

Treatment	mowing periods	stubble height	Description
T10H10	T10	H10	Cut and stubble 10 cm at 10 days of full head period in the first season
T10H30	T10	H30	Cut and stubble 30 cm at 10 days of full head period in the first season
T30H10	T30	H10	Cut and stubble 10 cm at 30 days of full head period in the first season
T30H30	T30	H30	Cut and stubble 30 cm at 30 days of full head period in the first season

Pre-germinated seeds were sown in seedbeds on 5 April 2021 and 23 March 2022, and 30-day-old seedlings were transplanted into the paddy field on 5 May 2021 and 22 April 2022. Seedlings were transplanted into the paddy soil with a hill spacing of 30 cm × 13.3 cm, with two seedlings per hill. During the first season of ripening, manual harvesting was carried out. During most of the rice growth period, the fields remained flooded until 7 days before MC ripening, except for about 10 days of drainage during the maximum tillering period (when the number of tillers reaches 80% of the expected number of panicles). In RC, the fields remained flooded until 10 days before maturity. Throughout the entire two-year growth season, weeds, diseases, and insects were strictly controlled.

### 2.2 Sampling and measurements

During the mature period of the ratoon season (except for the second row in each plot), 1 m^2^ (25 hills) of representative plant samples were taken to calculate grain yield and its constituent factors. The 1 m^2^ sample was divided into D2 (ears regenerated from the top 2nd section), D3 (ears regenerated from the top 3rd section), and D4 (ears regenerated from the lower section below the 3rd section) to calculate grain yield and its constituent factors separately. The empty spikelets were separated from the partially filled spikelets by winnowing. Three subsamples with 30 g filled spikelets and three subsamples with 2 g empty spikelets, along with all partially filled spikelets, were taken to count the number of spikelets (He et al., 2019).

Regeneration ability = Effective panicles per unit area during the ratoon season/Number of mother stems per unit area in the first season;

Regeneration ability at different nodes = Effective number of panicles per unit area at different nodes during the ratoon season/Number of mother stems per unit area in the head season.

Two rice plants were selected to measure the number and length of regenerated buds at 3 days, 9 days, 15 days, heading, and maturity periods after mowing. After washing, the regenerated buds from each node were peeled off, and the number of regenerated live buds (total number of buds with a length of ≥1 cm) and axillary bud length from the two sampled plants in D2 (second node from the top), D3 (third node from the top), and D4 (lower node below the third node of the stem). The regeneration rate and axillary bud length were calculated using the following formula:

Regeneration rate (%) = Number of live buds at each node/Number of mother stems × 100%; Axillary bud length (cm) = Sum of axillary bud lengths at each node/Number of buds.

Immediately after harvesting the first season crops, take their stubbles (except for the second row) and continuously take 12 hills (3 from each community × 4) Measure the fresh and dry weight of the rice stubble, then blanch at 105 °C for half an hour, and then dry it in a constant temperature drying oven at 80 °C to constant weight. Crush the dried rice stubble sample, pass it through a 100-mesh sieve, and divide it into two parts. One part was used to measure the organic carbon and nitrogen concentrations using an element analyzer (vario ISOTOPE cube, Elementar Trading Co., Ltd., Germany); the other part was used to determine the content of soluble sugars, starch, and non-structural carbohydrates (NSC) (He et al., 2019).

### 2.3 Data analysis

Variance analysis was performed using IBM SPSS Statistics version 22.0, and the means of treatments were compared based on the least significant difference (LSD) test at the 0.05 probability level. Statistical analysis and graphical representation of data were performed using GraphPad Prism version 9.0 software.

## 3 Results

### 3.1 Growth duration

The phenological data of various growth periods of rice are shown in **Table 3**. The reproductive periods of MC and RC are 120 days–145 days and 78 days–102 days, respectively, and the total reproductive period of RC was 53%–85% of MC. Under the same mowing period and conditions, the duration of RC increases with the decrease in stubble height. Under the same stubble height conditions, the duration of RC increases with the delay of the mowing period.

**Table 3:** Growth periods and durations of main and ratoon crops under different mowing periods and stubble height treatments.

Year	Treatment	Mowing period	Stubble height	Main crop season				Ratoon crop season		
				SW-TP	TP-HD	HD-MH	Main crop duration (d)	MH-HD	HD-RH	Ratoon crop duration(d)
2021	T10H10	T10	H10	31	79	10	120	49	53	102
	T10H30	T10	H30	31	79	10	120	41	43	84
	T30H10	T30	H10	31	79	30	140	47	57	104
	T30H30	T30	H30	31	79	30	140	36	57	93
2022	T10H10	T10	H10	31	84	10	125	45	43	88
	T10H30	T10	H30	31	84	10	125	36	42	78
	T30H10	T30	H10	31	84	30	145	46	47	93
	T30H30	T30	H30	31	84	30	145	35	47	82

Note: SW, the date of sowing; TP, the date of transplanting; HD, the date of heading; MH, the date of harest of main crop. RH:the date of harvest of ratoon crop.

### 3.2 Grain yield and composition of ratoon crops under different treatments

The grain yield under different treatments during the 2021 ratoon season was lower than that in 2022. The analysis of variance results showed that T had a significant impact on grain yield, panicles, filled grain rate, and 1,000-grain weight (p <0.01), while H had a significant impact on grain yield, panicles, spikelet panicle, and filled grain rate (P <0.01). Under the same stubble height conditions, the grain yield of T10 was higher than that of T30. Under the same mowing conditions, the grain yield of H30 was higher than that of H10. The highest grain yield was observed during the ratoon season under T10H30 treatment, ranging from 7,530 kg hm^−2^ in 2021 to 9,466 kg hm^−2^ in 2022. Compared with T30H30, T10H10 and T10H30 increased production by 48.1%, 41.7%, and 73.1%, 65.2%, respectively in the two years, while T30H10 reduced production by 30.9% and 19.5%, respectively.

Early mowing can improve all three factors of grain yield. However, higher stubble height significantly increased the number of panicles, while low stubble significantly increased the number of spikelets per panicle. On average, under the same mowing conditions, the number of panicles in H30 increased by 44.61% compared to H10, while the number of spikelets per panicle decreased by 27.76% compared to H10. The height of stubble had no significant effect on the filled grain rate. However, under different mowing periods, the 1,000-grain weight showed opposite trends under H30 and H10 treatment conditions.

### 3.3 Dry weight and fresh in stubble of MC

**Figure 2:**
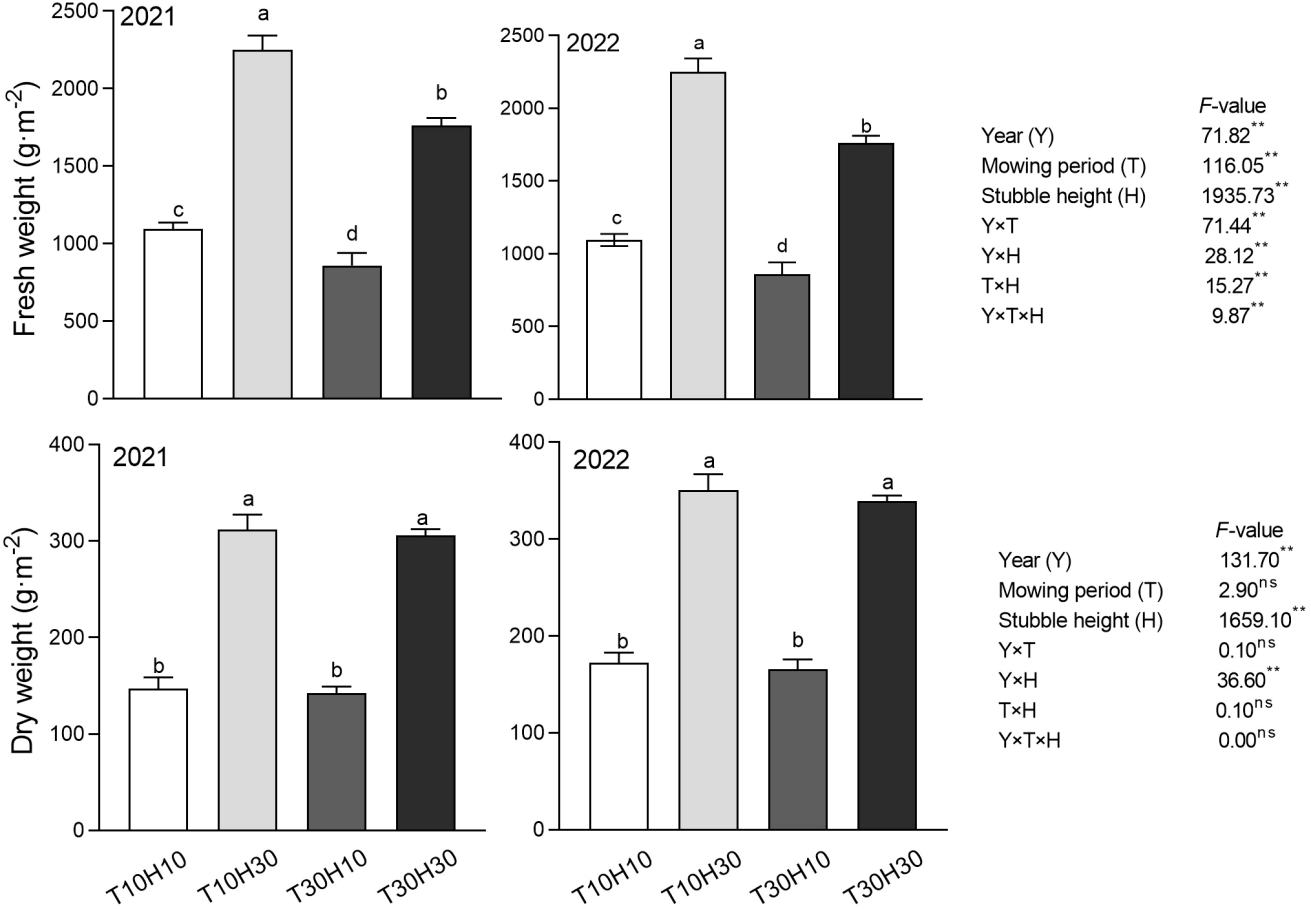
The fresh and dry weight under different mowing period and stubble heights in 2021 and 2022. Different lowercase letters denote statistical differences among four nitrogen treatments according to LSD test (0.05). Data are mean ± standard error. T10H10, Cut 10 days after heading in the first season and leave stubble height of 10 cm; T10H30, Cut 10 days after heading in the first season and leave stubble height of 30 cm; T30H10, Cut 30 days after heading in the first season and leave stubble height of 10 cm; T30H30, Cut 30 days after heading in the first season and leave stubble height of 30 cm. ** indicates significant effect at 0.01 level and ns indicates no significant effect.

Overall, the fresh weight of stubble in 2021 was higher than in 2022, while the dry weight of stubble in 2021 was lower than that in 2022 (**Figure 2**). Under the same stubble height conditions, the fresh weight of T10 stubble was greater than that of T30, while there was no significant difference in dry weight between T10 and T30 stubble. It was evident that the dry and fresh weights of high stubble height were significantly higher than those of low stubble height. Compared with T30H30, the two-year dry and fresh weights of T10H30 increased by 1.9%, 3.6%, 33.2%, and 4.0%, respectively, while the two-year dry and fresh weights of T10H10 decreased by 51.6%, 47.3%, 38%, and 47.2%, respectively. The two-year dry and fresh weights of T30H10 decreased by 53.4%, 49.2%, 53.4%, and 49.3%, respectively.

### 3.4 Nitrogen, Organic carbon content, and C/N of stubble

**Figure 3:**
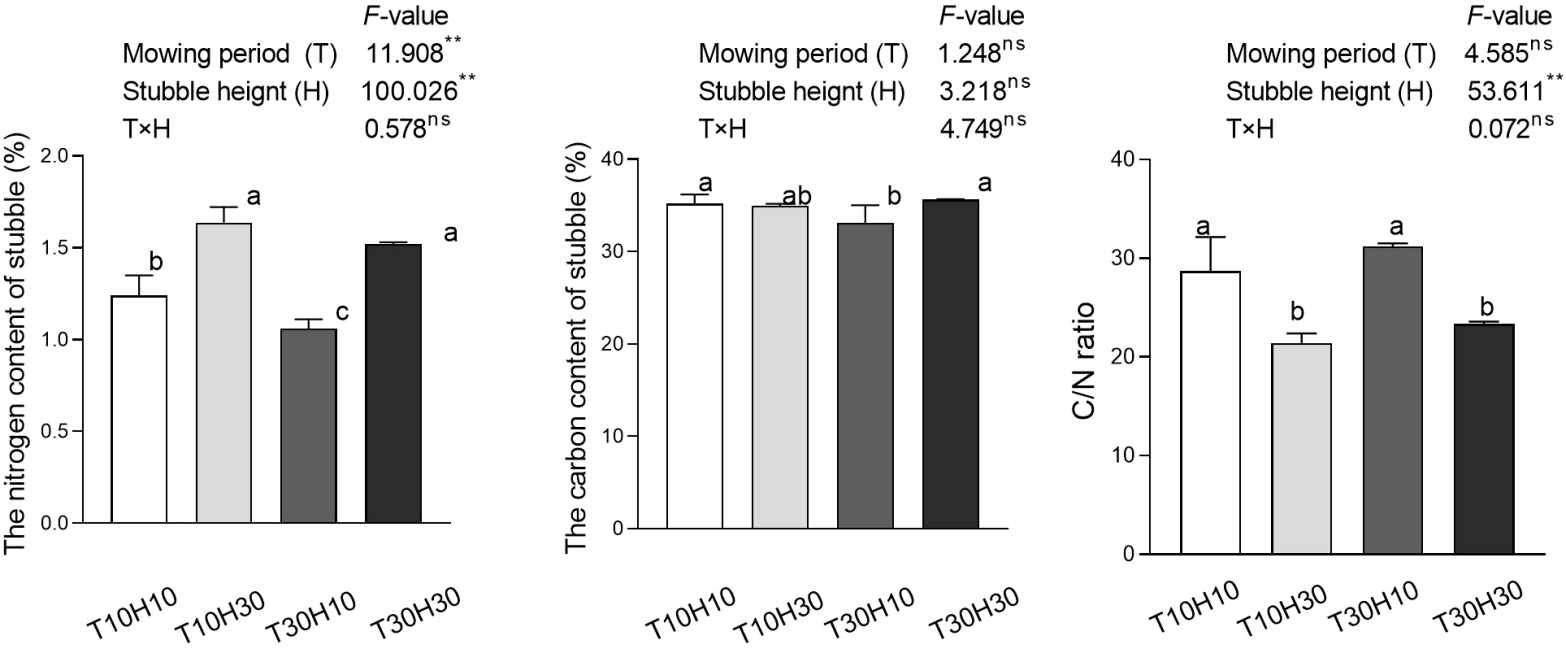
The nitrogen (N), carbon (C) content, and C/N of stubble under different mowing period and stubble heights in 2021 and 2022. Different lowercase letters denote statistical differences among four nitrogen treatments according to LSD test (0.05). Data are mean ± standard error. T10H10, Cut 10 days after heading in the first season and leave stubble height of 10 cm; T10H30, Cut 10 days after heading in the first season and leave stubble height of 30 cm; T30H10, Cut 30 days after heading in the first season and leave stubble height of 10 cm; T30H30, Cut 30 days after heading in the first season and leave stubble height of 30 cm. ** indicates significant effect at 0.01 level and ns indicates no significant effect.

The results of the analysis of variance showed that T and H had a significant impact on the nitrogen content of stubble, while the C/N ratio of stubble was only affected by H (**Figure 3**). Under the same stubble height conditions, the nitrogen content in T10 stubble was significantly higher than that in T30, while there was no significant difference in the C/N ratio of stubble. Under the same mowing period conditions, the nitrogen content in H30 stubble remained higher than in H10, while the C/N ratio in H30 stubble was significantly lower than in H10. Compared with T30H30, the nitrogen content of T10H30 stubble increased by 7.6%, while T10H10 and T30H10 decreased by 18.4% and 30.3%, respectively. Compared with T30H30, the organic carbon content of stubble in T10H10, T10H30, and T30H10 decreased by 1.2%, 1.8%, and 6.8%, respectively. Compared with T30H30, T10H10 and T30H10 increased rice stubble C/N by 22.9% and 33.6%, respectively, while T10H30 decreased by 8.3%.

### 3.5 Sugar, starch, and NSC content of stubble

**Figure 4:**
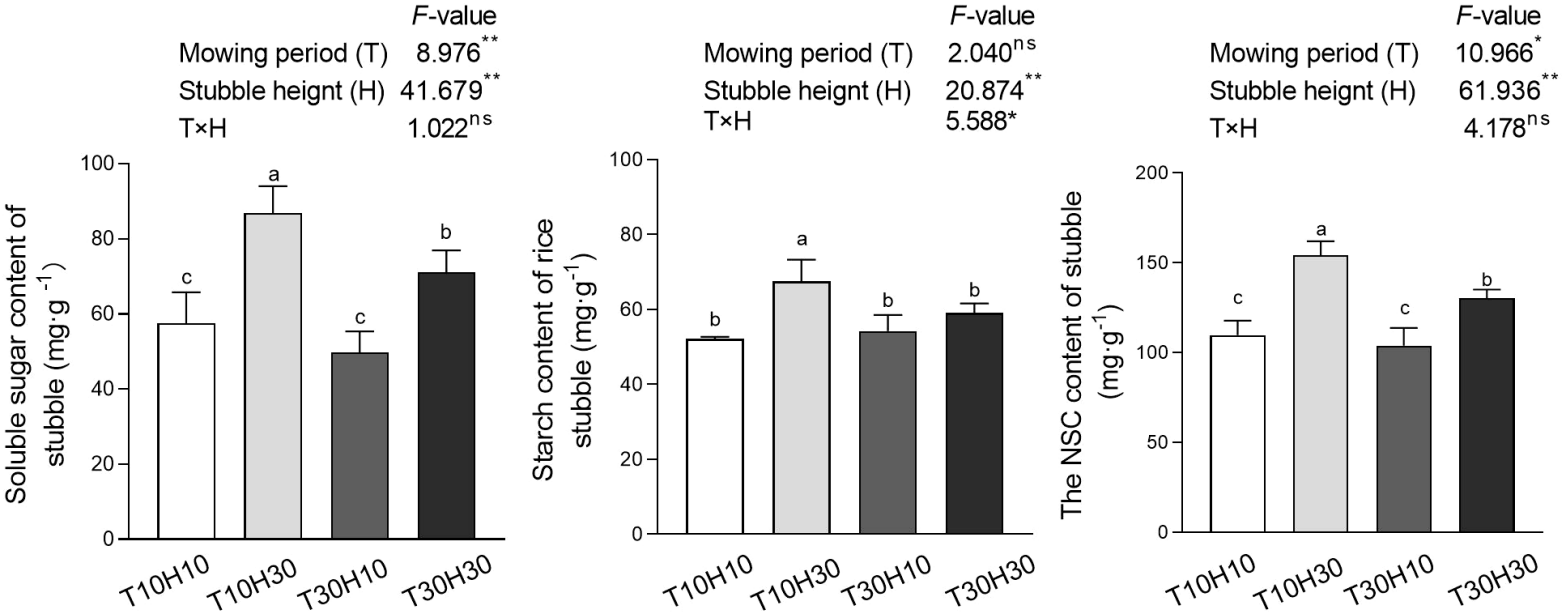
Effects of mowing period and stubble height on soluble sugar, starch, and NSC content of stubbles during the mowing period. Different lowercase letters denote statistical differences among four nitrogen treatments according to LSD test (0.05). Data are mean ± standard error. T10H10, Cut 10 days after heading in the first season and leave stubble height of 10 cm; T10H30, Cut 10 days after heading in the first season and leave stubble height of 30 cm; T30H10, Cut 30 days after heading in the first season and leave stubble height of 10 cm; T30H30, Cut 30 days after heading in the first season and leave stubble height of 30 cm.

The analysis of variance results showed that the effects of T and H on rice stem NSC and soluble sugar content reached a significant level, while only H had a significant impact on starch content (**Figure 4**). The influence trend of different treatments on the NSC and soluble sugar content of stubble was consistent. Under high stubble height conditions, the NSC and soluble sugar content of stubble in T10 were significantly higher than those in T30, while under low stubble height conditions, the difference between the two was not significant. Under the same mowing period conditions, the content of NSC and soluble sugars in H30 stubble remained higher than that in H10. The starch content of T10H30 stubble was significantly higher than the other three.

### 3.6 Regeneration rate of different nodes

**Figure 5:**
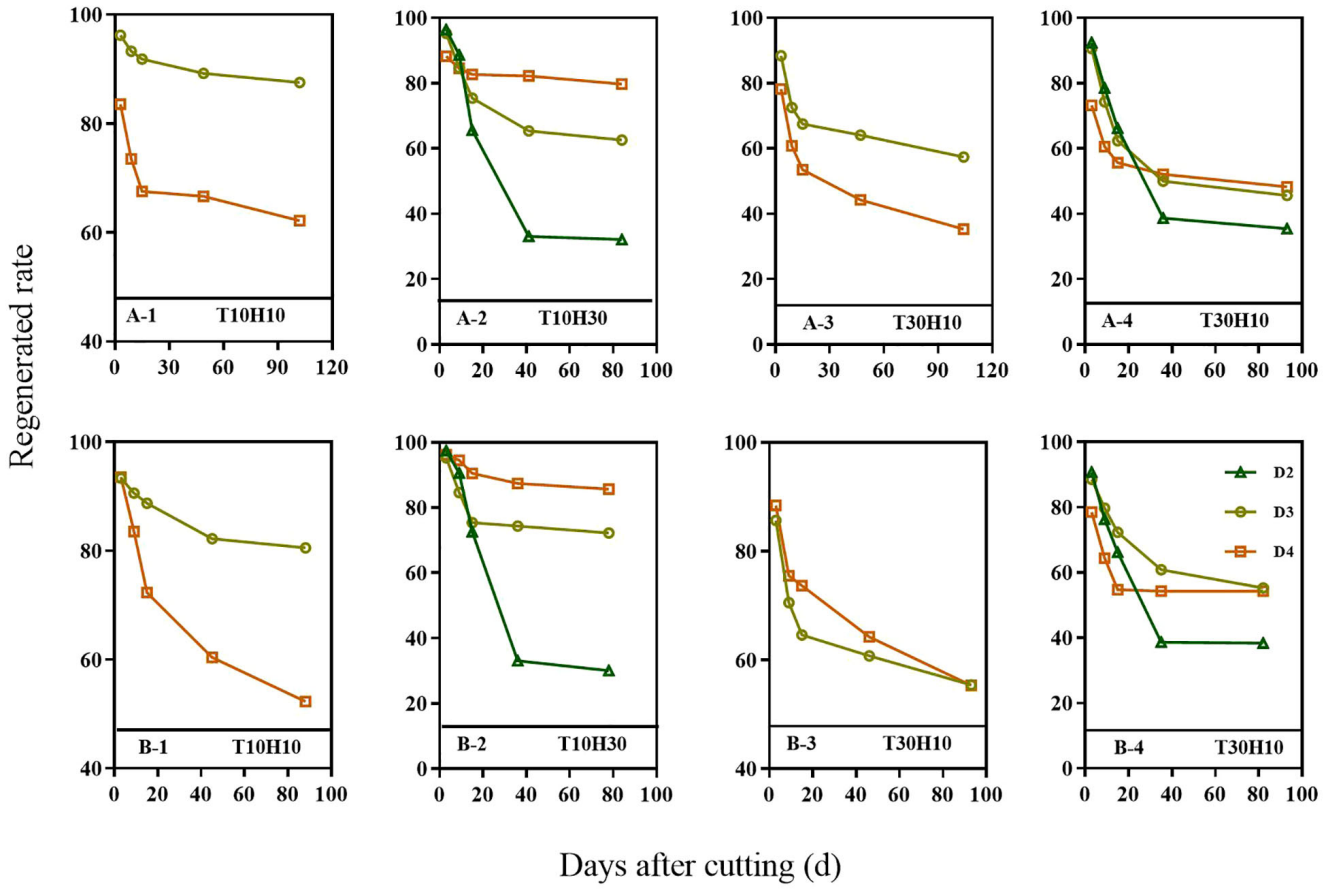
Regeneration rate in 2nd, 3rd, and 4th nodes (D2, D3, and D4) from the top of ratoon crop under different mowing periods and stubble heights in 2021 (A–X) and 2022 (B–X). T10H10 **(A-1, B-1)**, Cut 10 days after heading in the first season and leave stubble height of 10 cm; T10H30 **(A-2, B-2)**, Cut 10 days after heading in the first season and leave stubble height of 30 cm; T30H10 **(A-3, B-3)**, Cut 30 days after heading in the first season and leave stubble height of 10 cm; T30H30 **(A-4, B-4)**, Cut 30 days after heading in the first season and leave stubble height of 30 cm.

As the mowing period progressed, the regeneration rate of different treatments and nodes showed a downward trend (**Figure 5**). Overall, the regeneration rate of the upper node was higher than that of the lower node, and it was found that the upper node had a certain inhibitory effect on the growth and development of the lower node. Compared with T30H30, the regeneration rate of T10H30 at different nodes (D2, D3, D4) improved over the past two years. Compared with T30H30, the two-year regeneration rate of T10H10 D3 and D4 nodes improved, while the two-year regeneration rate of T30H10 D3 and D4 nodes has decreased.

### 3.7 Regeneration bud length of different nodes

**Figure 6:**
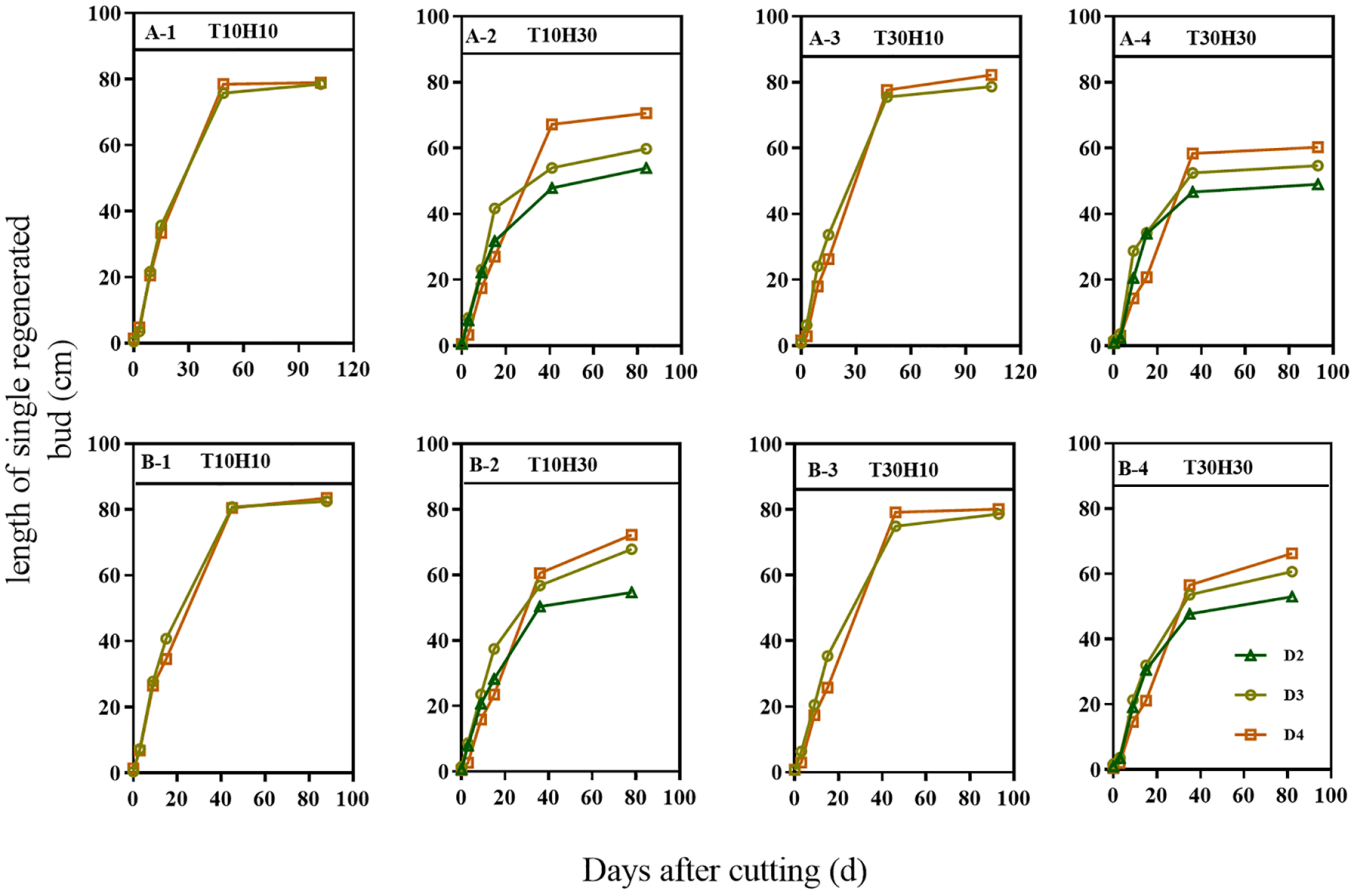
Bud length of regenerated tillers in 2nd, 3rd, and 4th nodes (D2, D3, and D4) from the top of ratoon crop under different mowing periods and stubble heights in 2021 (A–X) and 2022 (B–X). T10H10 **(A-1, B-1)**, Cut 10 days after heading in the first season and leave stubble height of 10 cm; T10H30 **(A-2, B-2)**, Cut 10 days after heading in the first season and leave stubble height of 30 cm; T30H10 **(A-3, B-3)**, Cut 30 days after heading in the first season and leave stubble height of 10 cm; T30H30 **(A-4, B-4)**, Cut 30 days after heading in the first season and leave stubble height of 30 cm.

As the mowing period progressed, the length of regenerated buds at different nodes of different treatments showed an upward trend (**Figure 6**). Overall, the length of regenerated buds at the lower node was greater than that at the upper node. Different mowing periods had no significant impact on the growth of regenerated seedlings at different nodes during the ratoon season, while different stubble heights have a significant impact on the growth dynamics of regenerated seedlings at different nodes during the ratoon season. The growth dynamics of regenerated seedlings in D3 and D4 sections were basically consistent with low stubble retention treatment; Under high stubble retention treatment, the growth rate of regenerated seedlings in D2 and D3 nodes was higher than that in D4 from 3 days to 15 days of mowing, and at the mature period, the regenerated seedlings in D4 nodes were greater than those in D2 and D3.

### 3.8 Regeneration ability

**Figure 7:**
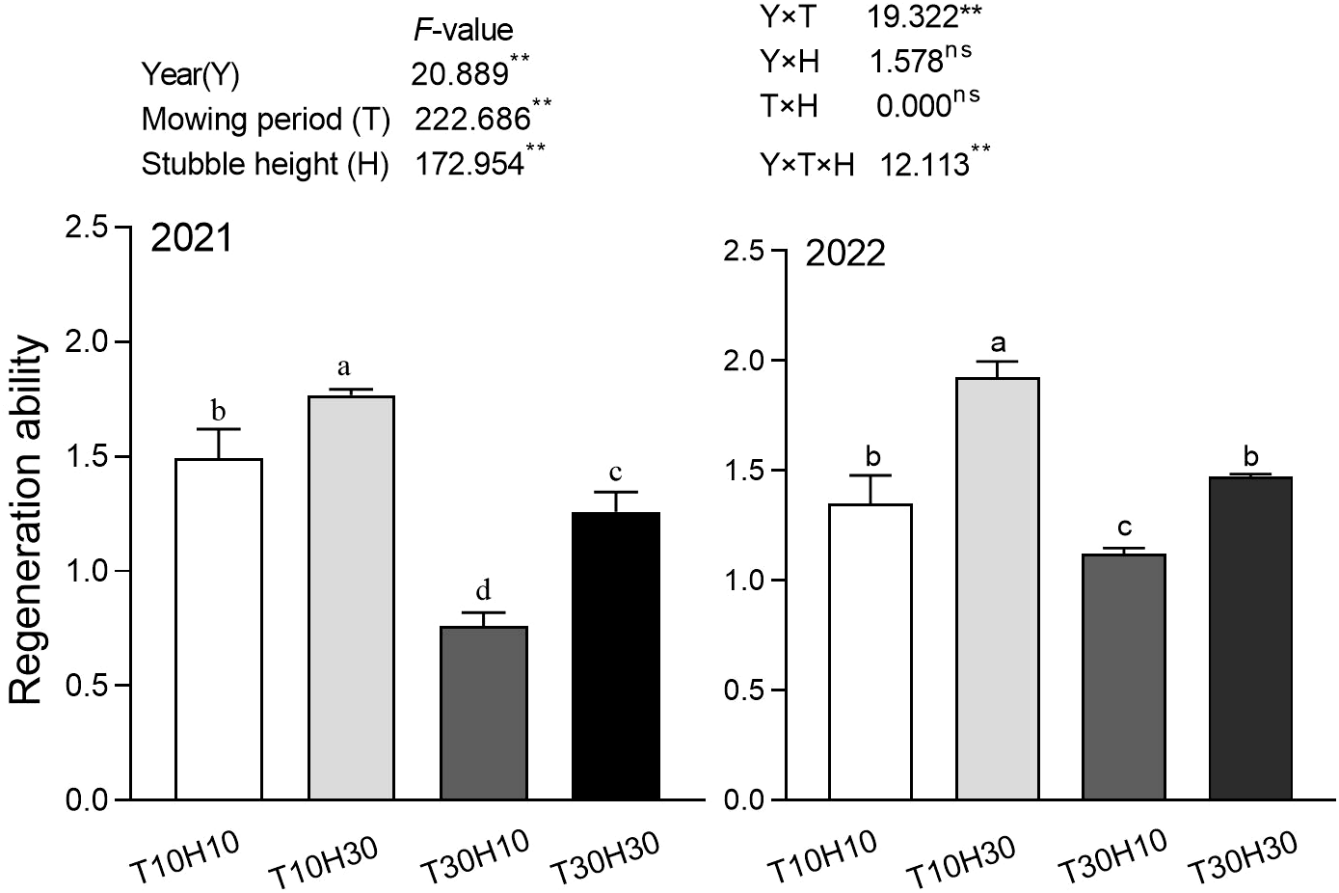
The effect of mowing period and stubble height treatment on the regeneration ability of regenerated crops. Different lowercase letters denote statistical differences among four nitrogen treatments according to LSD test (0.05). Data are mean ± standard error. T10H10, Cut 10 days after heading in the first season and leave stubble height of 10 cm; T10H30, Cut 10 days after heading in the first season and leave stubble height of 30 cm; T30H10, Cut 30 days after heading in the first season and leave stubble height of 10 cm; T30H30, Cut 30 days after heading in the first season and leave stubble height of 30 cm. ** indicates significant effect at 0.01 level and ns indicates no significant effect.

The results of the analysis of variance showed that Y, T, and H all had a significant impact on regenerative power (**Figure 7**). Under the same stubble height conditions, the ratoon force of T10 was significantly higher than that of T30. Under the same mowing period conditions, H30 had significantly higher regeneration ability than H10. We also found that the average regenerative power of T10 was significantly higher than that of H30. Compared with T30H30, the two-year ratoon capacity of T10H10 and T10H30 increased by 19.0%, 8.5%, and 40.5%, 30.6%, respectively, while the two-year ratoon capacity of T30H10 decreased by 39.4% and 23.9%, respectively.

### 3.9 Correlation analysis

**Figure 8:**
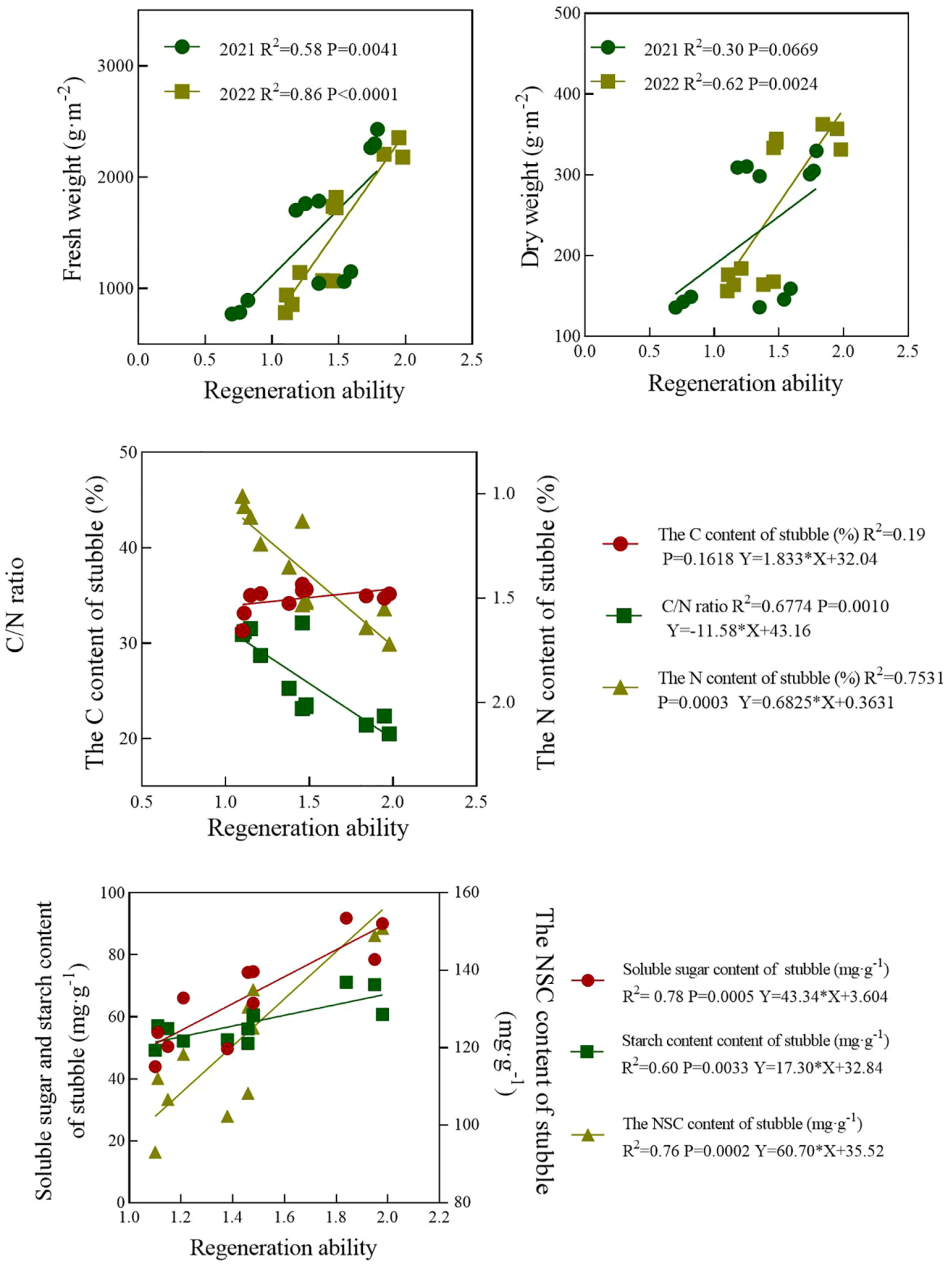
Correlation between regeneration ability of regenerated crops and fresh dry weight, C/N ratio, and NSC content of stubbles.

In 2021 and 2022, there was a significant positive correlation between ratoon capacity and fresh weight of stubbles, with correlation coefficients (R^2^) of 0.58 and 0.86 for the two years, respectively (**Figure 8**). The regeneration ability was significantly positively correlated with the dry weight of stubble, but the correlation was weaker compared to the fresh weight of stubble, with correlation coefficients (R^2^) of 0.30 and 0.62 for the two years, respectively. There was a positive correlation between regeneration ability and organic carbon and nitrogen content in stubbles, but the correlation between regeneration ability and organic carbon content was not significant. There was a highly significant negative correlation between regeneration ability and stubble C/N, with an R^2^ of 0.6774. There was a highly significant positive correlation between the regeneration ability and the content of soluble sugar, starch, and NSC in stubbles, with correlation coefficients (R^2^) of 0.78, 0.60, and 0.76, respectively.

### 3.10 Nitrogen accumulation and allocation of stubble and RC

**Figure 9:**
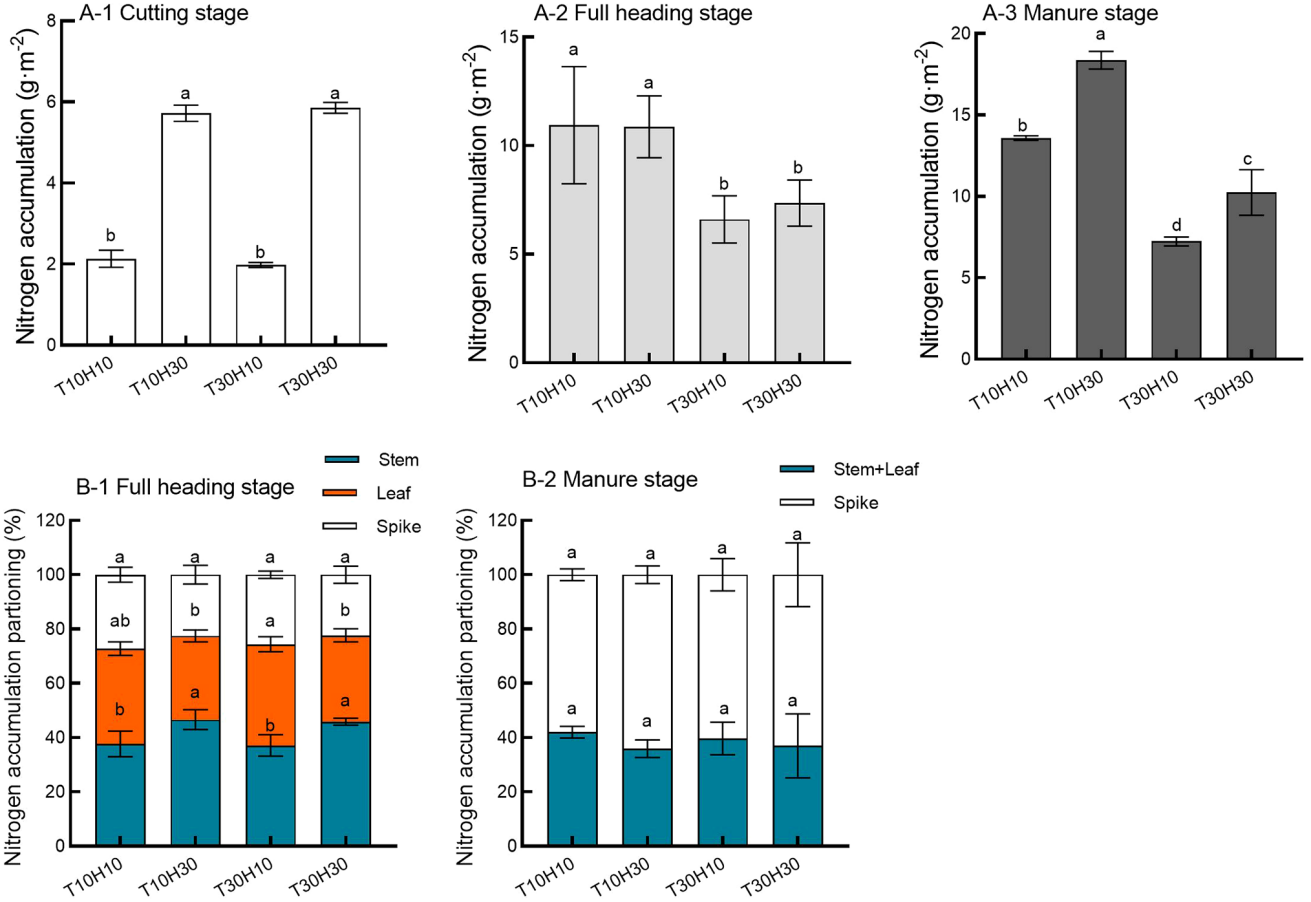
Effects of different mowing periods and stubble height on nitrogen accumulation and allocation in ratoon rice (**A-1** cutting stage, **A-2** full heading stage, **A-3** Manure stage, **B-1** full heading stage, **B-2** Manure stage). Different lowercase letters denote statistical differences among four nitrogen treatments according to LSD test (0.05). Data are mean ± standard error. T10H10, Cut 10 days after heading in the first season and leave stubble height of 10 cm; T10H30, Cut 10 days after heading in the first season and leave stubble height of 30 cm; T30H10, Cut 30 days after heading in the first season and leave stubble height of 10 cm; T30H30, Cut 30 days after heading in the first season and leave stubble height of 30 cm.

The conditions of different mowing periods and stubble height have varying degrees of impact on the nitrogen accumulation of regenerated crops during the mowing period, including rice stubble, full heading period, and mature period. During the mowing period, the accumulation of nitrogen in rice stubble was significantly higher in high stubble than in low stubble (**Figure 9A-1**). Under the same stubble height conditions, the effect of mowing period on the accumulation of stubble nitrogen was not significant (**Figure 9A-1**). Under the same mowing period conditions, there was no significant difference in the accumulation of nitrogen in regenerated crops during the full heading period due to the height of the stubble (**Figure 9A-2**). Under the same stubble height conditions, the nitrogen accumulation during the full heading period of regenerated crops harvested in advance was significantly higher than that of normal harvesting (**Figure 9A-2**). Under the same mowing period conditions, high retention stubble significantly increased the accumulation of nitrogen in mature regenerated crops compared to low retention stubble (**Figure 9A-3**). Under the same stubble height conditions, the nitrogen accumulation during the mature period of early mown regenerated crops was significantly higher than that of normal mown (**Figure 9A-3**).

There was a significant difference in the proportion of nitrogen distribution in different organs of regenerated crops during the full heading period between different mowing periods and stubble heights. Compared with T30H30, the proportion of nitrogen in the stem increased by 1.6%, while T10H10 and T30H10 decreased by 21.8% and 19.1%, respectively (**Figure 9B-1**). Compared with T30H30, the proportion of nitrogen in the leaves of T10H30 decreased by 2.8%, while T10H10 and T30H10 increased by 10.4% and 17.4%, respectively (**Figure 9B-1**). There was no significant effect of different mowing periods and stubble heights on the proportion of nitrogen allocation during the mature period of regenerated crops. Compared with T30H30, T10H30 increased the proportion of nitrogen elements in the panicle by 1.6%, while T10H10 and T30H10 decreased by 8.1% and 4.3%, respectively (**Figure 9B-2**).

## 4 Discussion

### 4.1 Early mowing and high stubble height can achieve higher ratooned grain yield

The first season cutting period and stubble height are important agronomic measures for determining RC grain yield. Overall, compared with H10, H30 showed an increase in the number of panicles and the filled grain rate, while spikelet panicle and 1,000-grain weight decreased to varying degrees, resulting in a final increase of 13.94%–44.80% (**Table 4**). This may be due to the high stubble height retaining more regenerated buds, especially the second node buds (D2). At this time, over 90% of 14C assimilates accumulate in the stem sheath, mainly distributed in the first and second internodes of the upper part, with less than 10% of assimilates allocated to other internodes. Therefore, D2 significantly contributes to the grain yield of the ratoon season under H30 treatment. On the other hand, under high stubble conditions, plants can have greener and more photosynthetic leaves, and the NSC in the rice stubble provides more carbohydrates for the plant (Nakano et al., 2020).We also observed an interesting phenomenon: compared to H10, H30 increased the grain setting rate of rice in the ratoon season (**Table 4**), which we believe is likely related to canopy temperature. Studies indicated that the average daily temperature for the growth of high retention staked plants was 0.9 °C higher than that of low retention stakes from tasseling to 40 days after tasseling. Obviously, when the height of the staking was reduced, rice spiked later and could not reach the appropriate temperature required for seed filling, which seriously affected seed filling. Low stubble height increases the number of grains per spike, mainly by reducing the growth point of axillary buds and increasing the number, size, and growth period of panicles starting from the basal node (Harrell et al., 2009; Xu et al., 2021). Some scholars believe that the grain yield potential of low stubble height is higher than that of high stubble height. However, the stability of low stubble height grain yield is lower, and its effectiveness depends on the local climate (Santos et al., 2003).

**Table 4:** Grain yield and composition of different nodes of regenerated crops under mowing period and stubble height treatment.

Year	Mowing period	Stubble height	Grain grain yield (kg·hm^-2^)	Panicles m^-2^	Spikelet panicle^-1^	Filled grain rate (%)	1000-grain weight (g)
2021	T10	H10	6443.93b	293.00c	154.83b	58.23a	24.07a
		H30	7342.53a	437.17a	118.77c	58.83a	23.20b
	T30	H10	3004.97d	243.67d	169.83a	35.8b	22.07c
		H30	4351.33c	333.50b	115.33c	35.33b	24.00a
2022	T10	H10	8118.27b	297.67c	165.83a	67.17b	23.60a
		H30	9466.37a	474.33a	115.00c	71.67a	23.00b
	T30	H10	4610.87d	261.33d	141.00b	48.10d	23.40ab
		H30	5729.03c	347.67b	105.70c	53.50c	23.03b
*F*-value	Year(Y)		270.866^**^	13.035^**^	3.777	673.510^**^	0.007
	Mowing period (T)		1101.335^**^	239.846^**^	0.027	1711.534^**^	12.449^**^
	Stubble height (H)		130.721^**^	593.296^**^	24.332^**^	25.346^**^	0.683
	Y×T		3.907	0.240	0.360	18.744^**^	2.497
	Y×H		0.288	2.020	1.106	23.381^**^	14.563^**^
	T×H		0.280	50.268^**^	1.004	0.002	34.753^**^
	Y×T×H		2.705	3.113	0.046	0.926	35.736^**^

Different lowercase letters denote statistical differences among grain yield at different nodes under the same treatment for each year according to LSD test (0.05). Year (Y), Mowing period (T), Stubble height (H), Y × T, Y × H, T × H, Y × T× H refer to the year, mowing period, stubble height, and their interactions, respectively. * and ** indicate significant differences at the 0.05 and 0.01 levels, respectively. Lowercase letters following the data within the same column refer to significant differences (P <0.05).

In this study, compared with T30, T10 significantly increased the number of panicles, spikelets per panicle, and the filled grain rate of ratoon rice, while having a relatively small impact on 1,000-grain weight, resulting in a final increase of 65.24%–114.44% (**Table 4**). The main reason for the increase in T10 production may be that NSC in the stubble was not completely transferred to the grains at early mowing, and most of it still accumulated in the nutrient organs (Yoshinaga et al., 2013). On the other hand, late harvesting party in the first season of ratoon rice led to a delay in the growth period in the second season, and the late period of reproduction did not have enough heat due to the cool weather, which led to irregular tasseling in the second season and low grain yield (Albacete et al., 2014).

### 4.2 Ways to increase the grain yield of ratoon season through early mowing and higher stubble height

According to reports, the grain yield of RC depends on the nutritional status of MC stubble, such as dry weight, soluble sugar, and NSC content. The specific analysis of the indicators related to the characteristics of stubble will not be conducted here. These indicators themselves have a significant correlation with the grain yield of the ratoon season, and the patterns presented are almost similar to the grain yield. For example, compared with T30, T10 was more conducive to improving the dry weight, nitrogen content, and NSC content of stubble, while H30 increased the amount of stubble characteristic indicators to varying degrees compared to H10. Possible reasons for this: On one hand, the heavier the dry weight of the stem and sheath, the more carbohydrates were left behind by the nutrient organs mainly composed of the stem and sheath, and the higher the content of carbohydrates (total sugars, soluble sugars, etc.) in the rice stubble (Ren et al., 2006). On the other hand, NSC in the stubble is the foundation for the early growth of ratoon rice. Early mowing results in incomplete transfer of NSC from the stem to the grain (the amount of transfer to the grain depends on the mowing period), and most of it still accumulates in the nutrient organs, which may be necessary for the growth of the second crop. In short, early mowing and higher stubble height can provide more carbohydrates for the growth of regenerated roots and buds. However, through summary and analysis, we found that the trend of grain yield changes also differs from the characteristics of stubble.

Analysis shows that the grain yield of the ratoon season was positively correlated with the dry weight of stubble but weakly correlated with the fresh weight (**Figure 8**). The effect of stubble height on the dry and fresh weight of stubble was more significant than that during the mowing period. This is worth noting, as it does not fully match the pattern of grain yield changes during the ratoon season. Analysis revealed that the grain yield of the ratoon season was positively correlated with the nitrogen content in stubble, independent of the organic carbon content, and negatively correlated with the C/N ratio (**Figures 3**, **8**), indicating that the nitrogen content in stubble was related to grain yield formation. Moreover, the average nitrogen content of stubble in high stubble height was 1.09 times higher than that of early mowing. Next, we analyzed the stubble NSC and found that the H30 stubble NSC and soluble sugar content were consistently higher than H10, and the average values of stubble NSC and soluble sugar content in higher stubble height were higher than those in early mowing (**Figure 4**). A pattern was found that under different treatments, the dry weight, nitrogen content, and NSC of stubble showed a consistent trend, and high stubble height retention had a greater impact on the above indicators than early mowing. However, early mowing has a direct impact on regeneration ability (**Figure 7**), and this impact is much greater than high stubble retention and normal mowing. The grain yield of the ratoon season seems to be more consistent with the variation pattern of regeneration ability, with the highest grain yield being achieved through early mowing. We think that the increase in grain yield during the ratoon season is not only influenced by the characteristics of stubble, but may also be more closely related to endogenous hormones. According to reports, thinning during the full heading period not only changes the transportation, distribution, and metabolism of assimilates in plants, but also alters the content of hormones and other physiologically active substances in plants (Griffiths et al., 2016). Studies indicated that sparse panicles during the full heading period of rice affect the germination and growth of axillary buds by breaking the apical dominance, altering the transportation and distribution of IAA, and the transcriptional expression of related genes (Arite et al., 2010). In short, there are different ways to increase grain yield between early mowing and high stubble. Early mowing increases grain yield due to changes in endogenous hormones, while high stubble stores more nutrients in stubble.However, we have a limited understanding of the relationship between endogenous hormone content and the regeneration rate of regenerated plants, which can serve as our next research direction. On the other hand, higher stubble height has a greater impact on nitrogen accumulation in early plants than early mowing and lower stubble height. However, during the full heading and mature periods, T10 has a much greater impact on nitrogen accumulation in plants than H30. Early mowing is beneficial for the accumulation of nitrogen in the late period of the ratoon season of ratoon rice, which may be another reason for increased grain yield.

### 4.3 The impact of climate and growth period on the grain yield of the ratoon season

The average grain yield in the ratoon season was 5.29 t ha^−1^ in 2021, significantly lower than in 2022 (6.98 t ha^−1^) (**Table 4**). The difference in the grain yield of the ratoon crop between 2021 and 2022 might be related to differences in solar radiation and total effective accumulated temperature during the ratoon season in the two years. The daily average solar radiation and total effective accumulated temperature from the harvest of the main crop to the maturity of the ratoon season were 14.15 MJ m^−2^d^−1^ and 1,348 °C in 2022, respectively, while in 2021 they were 13.48 MJ m^−2^d^−1^ and 1,204 °C, respectively. According to reports, there is a positive correlation between rice grain yield and daily effective accumulated temperature (Xie et al., 2016). Nagarajan et al. (2010) showed that when the daily average solar radiation was less than 21 MJ m^−2^d^−1^, there was a positive correlation between rice grain yield and daily average solar radiation. In our study, the ratoon capacity in 2022 was 1.11 times that of 2021, and the seed setting rate in 2022 was much higher than in 2021, which was 1.28 times higher. Perhaps it was because sufficient temperature and solar radiation are conducive to grain filling and maturation of regenerated panicles (He et al., 2019). Delaying the main crop mowing period and reducing the height of stubble extended the growth cycle of the ratoon season. However, studies indicate that delaying the growth cycle has a negative impact on climate factors, leading to lower accumulated temperature, accumulated sunshine hours, and accumulated rainfall during the ratoon season, thereby reducing the grain yield during the ratoon season (Qi et al., 2024a).

## 5 Conclusion

**Figure 10:**
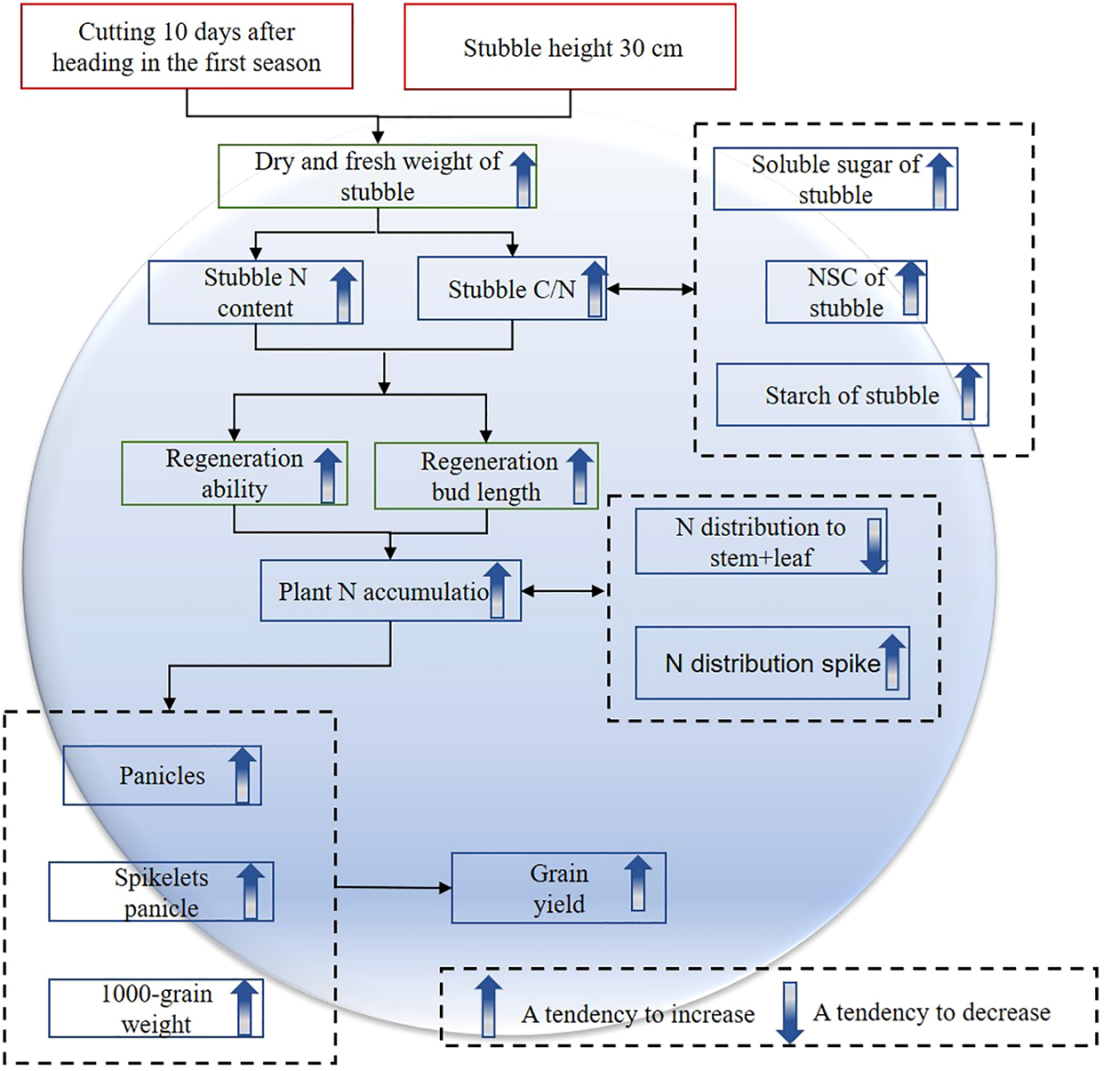
Path analysis of increasing rice grain yield under early mowing and high stubble retention conditions.

Compared to delayed harvesting and lower stubble retention, early harvesting and higher stubble retention are suitable for improving the grain yield of the ratoon season (**Figure 10**). Early mowing increases grain yield and the three components of grain yield to varying degrees. However, increasing the number of panicles with high stubble height reduces spikelet per panicle, but here the increase in source contributes more to the grain yield of the ratoon season than sink.

## Data Availability

The original contributions presented in the study are included in the article/supplementary material. Further inquiries can be directed to the corresponding authors.
